# Bat ticks revisited: *Ixodes ariadnae* sp. nov. and allopatric genotypes of *I. vespertilionis* in caves of Hungary

**DOI:** 10.1186/1756-3305-7-202

**Published:** 2014-04-27

**Authors:** Sándor Hornok, Jenő Kontschán, Dávid Kováts, Richárd Kovács, Dorottya Angyal, Tamás Görföl, Zsolt Polacsek, Zsuzsa Kalmár, Andrei D Mihalca

**Affiliations:** 1 Department of Parasitology and Zoology, Faculty of Veterinary Science, Szent István University, Budapest, Hungary; 2 Plant Protection Institute, Centre for Agricultural Research, Hungarian Academy of Sciences, Budapest, Hungary; 3 Department of Zoology and Animal Ecology, Szent István University, Gödöllő, Hungary; 4 Department of Evolutionary Zoology and Human Biology, University of Debrecen, Debrecen, Hungary; 5 Ariadne Caving Group, Pilis, Hungary; 6 Department of Zoology, Hungarian Natural History Museum, Budapest, Hungary; 7 Institute for Veterinary Medical Research, Centre for Agricultural Research, Hungarian Academy of Sciences, Budapest, Hungary; 8 Hungarian Karst- and Cave Research Society, Budapest, Hungary; 9 Department of Parasitology and Parasitic Diseases, Faculty of Veterinary Medicine, University of Agricultural Sciences and Veterinary Medicine, Cluj-Napoca, Romania

**Keywords:** *Ixodes*, Tick, Cave, Bat, Genotype

## Abstract

**Background:**

In Europe two ixodid bat tick species, *Ixodes vespertilionis* and *I. simplex* were hitherto known to occur.

**Methods:**

Bat ticks were collected from cave walls and bats in Hungary. Their morphology and genotypes were compared with microscopy and conventional PCR (followed by sequencing), respectively.

**Results:**

A year-round activity of *I. vespertilionis* was observed. Molecular analysis of the cytochrome oxidase subunit I (COI) gene of twenty ticks from different caves showed that the occurrence of the most common genotype was associated with the caves close to each other. A few specimens of a morphologically different tick variant were also found and their COI analysis revealed only 86-88% sequence homology with *I. simplex* and *I. vespertilionis*, respectively.

**Conclusions:**

The microenvironment of caves (well separated from each other) appears to support the existence of allopatric *I. vespertilionis* COI genotypes, most likely related to the distance between caves and to bat migration over-bridging certain caves. The name *I. ariadnae* sp. nov. is given to the new tick species described here for the first time.

## Background

In Europe, two ixodid bat tick species are known to occur [1]. The long-legged bat tick (*Ixodes vespertilionis*) has a broad host range and a worldwide distribution; accordingly, it was also reported from most of the European countries [2]. On the other hand, *I. simplex* is highly specialized to the bat *Miniopterus schreibersii*, and (although geographically widespread) it is usually found in low numbers during surveys [3]. This may be partly attributed to the difficulties in its diagnosis, which is not always clear [2]. The taxonomical relationship of the two bat tick species is also a matter of debate, as formerly they were classified as members of separate subgenera (*Eschatocephalus* and *Pomerantzevella*, respectively), which were later judged to be synonymous [4]. Furthermore, *I. vespertilionis* is usually collected from either bats [3] or cave walls [5], but *I. simplex* almost exclusively from bats [2,3,6].

Concerning the epidemiological significance of bat ticks, *I. vespertilionis* was shown to be present outside caves (e.g. cellars or attics of houses, tree holes: [3]). This species may also infest humans [7] and may carry bartonellae [5]. However, *I. simplex* is unlikely to feed on humans, and its vector potential remains to be elucidated.

Recently, during collections of ticks in Hungarian caves [5] it was observed, that some specimens are morphologically different from both *I. vespertilionis*, and *I. simplex*. Arthur [2] also reported the existence of bat ticks, which show intermediate features between *I. vespertilionis* and *I. simplex*. Therefore, the primary aim of the present study was to collect large numbers of ticks from caves, in order to clarify their taxonomical status on both morphological and molecular bases. It was also within the scope of the survey to obtain data on the ecology of bat ticks, i.e. their seasonality and the distribution of cave-related (allopatric) genotypes.

The cytochrome oxidase subunit I (COI) gene was chosen for molecular analysis, on account of its suitability as a DNA-barcode sequence for animal/tick species identification [8,9]. The COI gene allows phylogenetic studies, because it is conserved enough within species, shows variability among species, and there are numerous sequences of this gene from ixodid ticks already deposited in the GenBank.

## Methods

**Figure 1:**
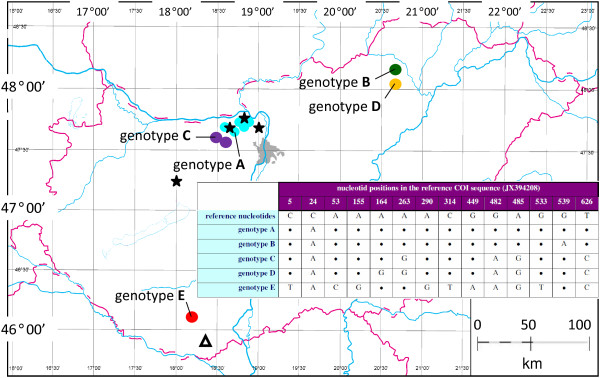
**Map of Hungary showing the geographical distribution of cave-related, allopatric *I. vespertilionis *genotypes (coloured dots), and collection sites of *I. ariadnae *sp. nov. (stars). *I. simplex *was removed from *Miniopterus schreibersii *in southern Hungary (triangle).** The table insert illustrates nucleotide differences between the genotypes according to a reference sequence with the highest similarity to the Hungarian isolates.

Tick collections of the present study were carried out in 2012-2013, and consisted of three parts. The main collection site included three caves (Legény Cave, Leány Cave and Ariadne Cave; entrances within 1 km; central coordinate: 47° 41′ 57.67″ N, 18° 50′ 39.24″ E) in the Pilis Mountains, that were visited repeatedly during the winter, spring and autumn months. A few ticks were also provided by speleologists from more distant locations selected randomly (i.e. seven caves in the Gerecse, Bükk and Mecsek Mountains: Figure 1).

Additionally, five ticks (three nymphs, one larva with unusual morphology; and one *I. simplex* nymph from *Miniopterus schreibersii*: Figure 1) removed from five bats during their mating period (August-September) were included in the present study. These animals were caught (as part of a monitoring program) at the entrance of caves between sunset and dawn, using standard Ecotone mist-nets (Gdynia, Poland) with 12 m length, 2.5 m height and 14 × 14 mm mesh [10,11]. Ticks were immediately put into and stored in 70% ethanol.

DNA extraction from ticks was carried out as described [12]. For barcoding a portion of the COI gene was amplified from these tick DNA samples with the universal primer pair LCO/HCO [13] in a concentration of 10 pmol/μl. PCR conditions were the following: 94°C, 5 min; then 35 cycles of 94°C, 40 sec, 44°C, 40 sec and 72°C, 1 min; and final extension at 72°C for 5 min. The length of PCR product was approximately 700 bps. After electrophoresis in 1.5% gel the bands were excised and purified by using the Wizard® SV Gel and PCR Clean-Up System (Promega, USA). Sequencing was done by Macrogen Inc. (Korea). Sequences were submitted to the GenBank (accession numbers KJ490305 for *I. simplex*, and KJ490307-KJ490311 for genotypes of *I. vespertilionis* designated A-E, respectively). Phylogenetic analysis was performed with Mega 5.2 program package, and the tree was constructed by Neighbor-Joining test.

Scanning micrographs were made in the Hungarian Natural History Museum (Budapest) with a HITACHI SN 2600 scanning electron microscope. All investigated specimens were sputter coated by golden-palladium.

### Ethical approval

Authorization for bat capture was provided by the National Inspectorate for Environment, Nature and Water (No. 14/2138-7/2011). Bat banding licence numbers are TMF-14/32/2010 and TMF-493/3/2005.

## Results

### Spatiotemporal distribution of *I. vespertilionis*

In the caves, altogether 535 ticks were collected: 527 *I. vespertilionis* (220 males, 119 females, 171 nymphs and 17 larvae), and eight specimens showing different morphology (only engorged females). Concerning *I. vespertilionis*, except for the summer months (with low tick activity) non-parasitic males outnumbered females in the populations. The presence of questing females on cave walls appeared to be equilibrated between the seasons (Table 1). However, the proportion of nymphs was more pronounced during the spring and autumn. Larvae were observed from March to June.

**Table 1:** **Collection data of *I. vespertilionis *** **in caves according to seasons**

	**Winter (6/6)**				**Spring (18/12)**				**Summer (3/3)**				**Autumn (4/4)**			
	**Larvae**	**Nymphs**	**Males**	**Females**	**Larvae**	**Nymphs**	**Males**	**Females**	**Larvae**	**Nymphs**	**Males**	**Females**	**Larvae**	**Nymphs**	**Males**	**Females**
Total number	-	5	23	7	16	114	160	92	1	-	3	3	-	52	34	17
Percentage	-	14%	66%	20%	4%	30%	42%	24%	14%	-	43%	43%	-	50%	33%	17%
Presence of bats	+++				++				+				++			

Next to each season the number of visists per number of caves is shown in brackets.

**Figure 2:**
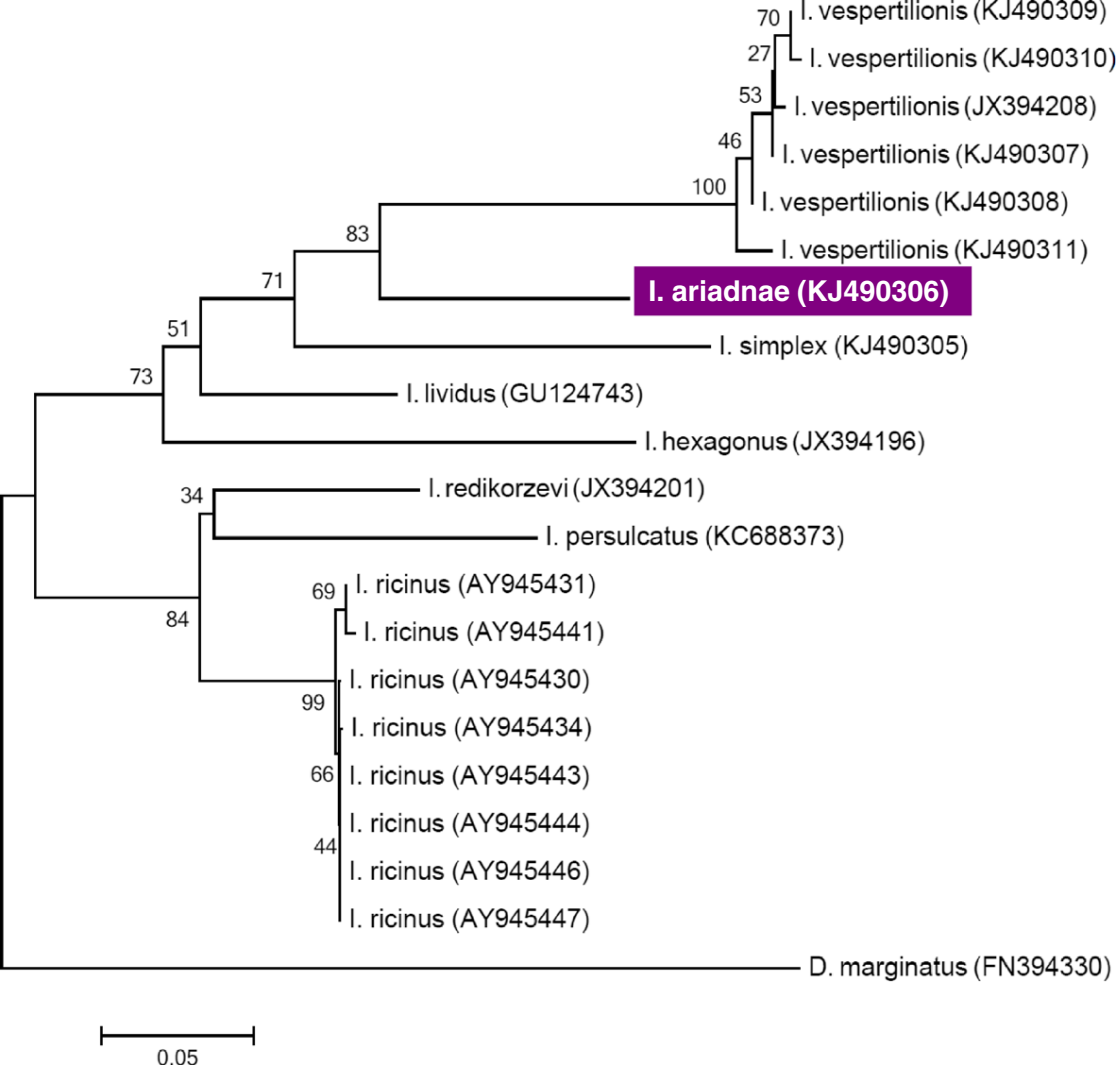
**Phylogenetic relationship of *I. ariadnae *** **sp. nov., *I. vespertilionis *** **genotypes and *I. simplex *** **inferred from COI gene analysis in the present study, in comparison with other *Ixodes *** **spp. and *Dermacentor marginatus *** **based on sequences deposited in the GenBank.**

Twenty ticks were selected for molecular analysis, including 15 specimens of *I. vespertilionis* (eight from the main collection sites, seven from other caves), four specimens with different morphology, and for comparison one *I. simplex* collected from *Miniopterus schreibersii*. In the three caves of the main collection site only genotype A was identified (corresponding to 8 tick specimens). This genotype was also found in two caves within 20 km westward, but not in any other, more distant caves. In two further caves within 40 km southwest genotype C was identified, and in three caves approx. 200 km to the east and to the south three other genotypes (B, D, and E: Figure 1). Genotypes A, B, C, D and E differed from the reference sequence (accession number JX394208) in one, two, five, six or eleven nucleotides, respectively (Figure 1). Their phylogenetic relationships with each other and further ixodid species occurring in Europe are shown on Figure 2.

### Description of *Ixodes ariadnae* Hornok sp. nov

#### Taxonomic summary

Phylum Arthropoda, class Arachnida, subclass Acari, family Ixodidae, genus *Ixodes*.

#### Diagnosis

Medium size (engorged females 6-7 mm) prostriate ticks. The legs long, palps short and hypostome medium length. Scutum rounded, broad hexagonal, posteriorly curved, with deep cervical grooves on engorged specimens. Hair covering sparse both dorsally and ventrally. Only engorged females, nymphs and a larva were collected. Male is unknown.

#### Type material and locality

##### Holotype

(1) female, from Leány Cave, Pilis Mountains, Hungary, (collected by D. Angyal, March 31, 2012).

##### Paratypes

(2) nymph, from *Myotis alcathoe*, Pisznice Cave, Gerecse Mountains, Hungary (collected by D. Kováts, August 25, 2012); (3)-(6) three females from Leány Cave and one female from Legény Cave, Pilis Mountains, Hungary, (collected by D. Angyal, March 31, 2012); (7)-(9) three females from Leány Cave, Pilis Mountains, Hungary, (collected by S. Hornok, March 24, 2013); (10) nymph from *Plecotus auritus* at Szopláki-ördöglyuk Cave, Pilis Mountains, Hungary, (collected by D. Kováts, April 6, 2012); (11) nymph from *Myotis blythii* at Szopláki-ördöglyuk Cave, Pilis Mountains, Hungary, (collected by D. Kováts, September 4, 2012); (12) larva from *Myotis alcathoe* at Alsópere, Bakony Mountains, Hungary, (collected by D. Kováts, July 27, 2013).

Holotype and paratypes (2), (4)-(6) are stored in alcohol and deposited at the Department of Parasitology and Zoology, Szent István University. Paratypes (8)-(10) are stored in alcohol and deposited in Soil Zoology Collection of the Hungarian Natural History Museum. Paratypes (3), (7), (11) and (12) were used for molecular phylogenetical comparison.

#### Morphology

Anal groove anterior to the anus (genus *Ixodes*).

Female (engorged): Length 6 mm. Legs long (tarsus I: 1.5 mm). Haller’s organ open, long, confluent, with 11 setae in three groups. On the basis capituli posterolaterally blunt flange, posterior edge with dorsal ridge (maximum width 0.5 mm). Palps short (0.44 mm), broad at the distinct joining of segments II-III. Hypostome of medium length (0.35 mm), with 10 rows of broad teeth, situated ventrally in four lines. Scutum rounded, broad hexagonal, with a shape index of 1.2. Idiosoma has sparse hair covering both dorsally and ventrally. Coxae are convex posteroexternally, with rounded surface. Genital pore between coxae III. Anal groove posteriorly divergent. Spiracle openings oval, with irregular outline.

Nymph (engorged): Length 3 mm. Legs moderately long (tarsus I: 0.75 mm). Basis capituli at maximum width 0.35 mm. Palps short (0.2 mm; segments II + III: 0.125 + 0.075 mm), broad at the distinct joining of segments II-III. Hypostome of medium length (0.13 mm). Scutum rounded, broad hexagonal (length: 0.72 mm, width: 0.6 mm, shape index 1.2). Idiosoma has sparse hair covering both dorsally and ventrally. Spiracles oval, with irregular outline.

#### Differential diagnosis

**Figure 3:**
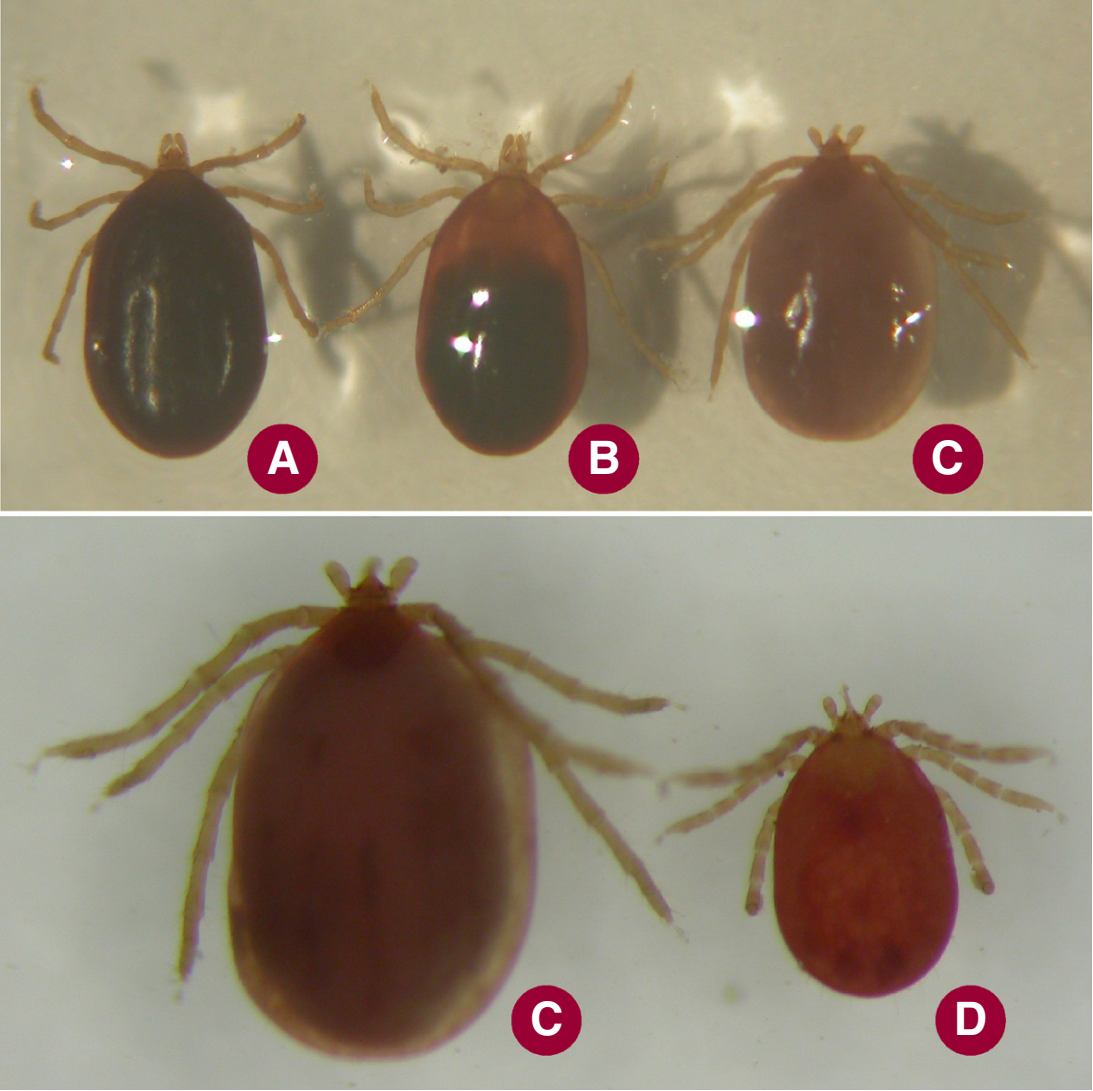
**Larva of *I. ariadnae *** **sp. nov. collected from *Myotis alcathoe *** **and showing apolysis (C), in comparison with *I. vespertilionis *** **larvae from the cave wall without (A) or with apolysis (B); and (D) *I. simplex *** **larva collected from *Miniopterus schreibersii*.**

**Figure 4:**
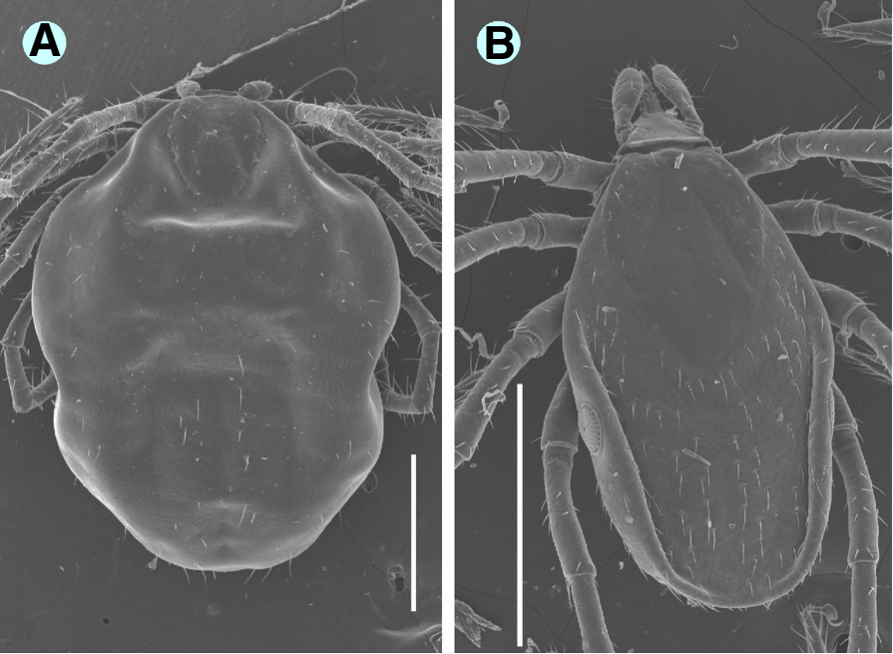
**Scanning electron microscopy (SEM) pictures of engorged *I. ariadnae *** **sp. nov. nymph (A) and unfed *I. vespertilionis *** **nymph (B) (bars: 1 mm).**

**Figure 5:**
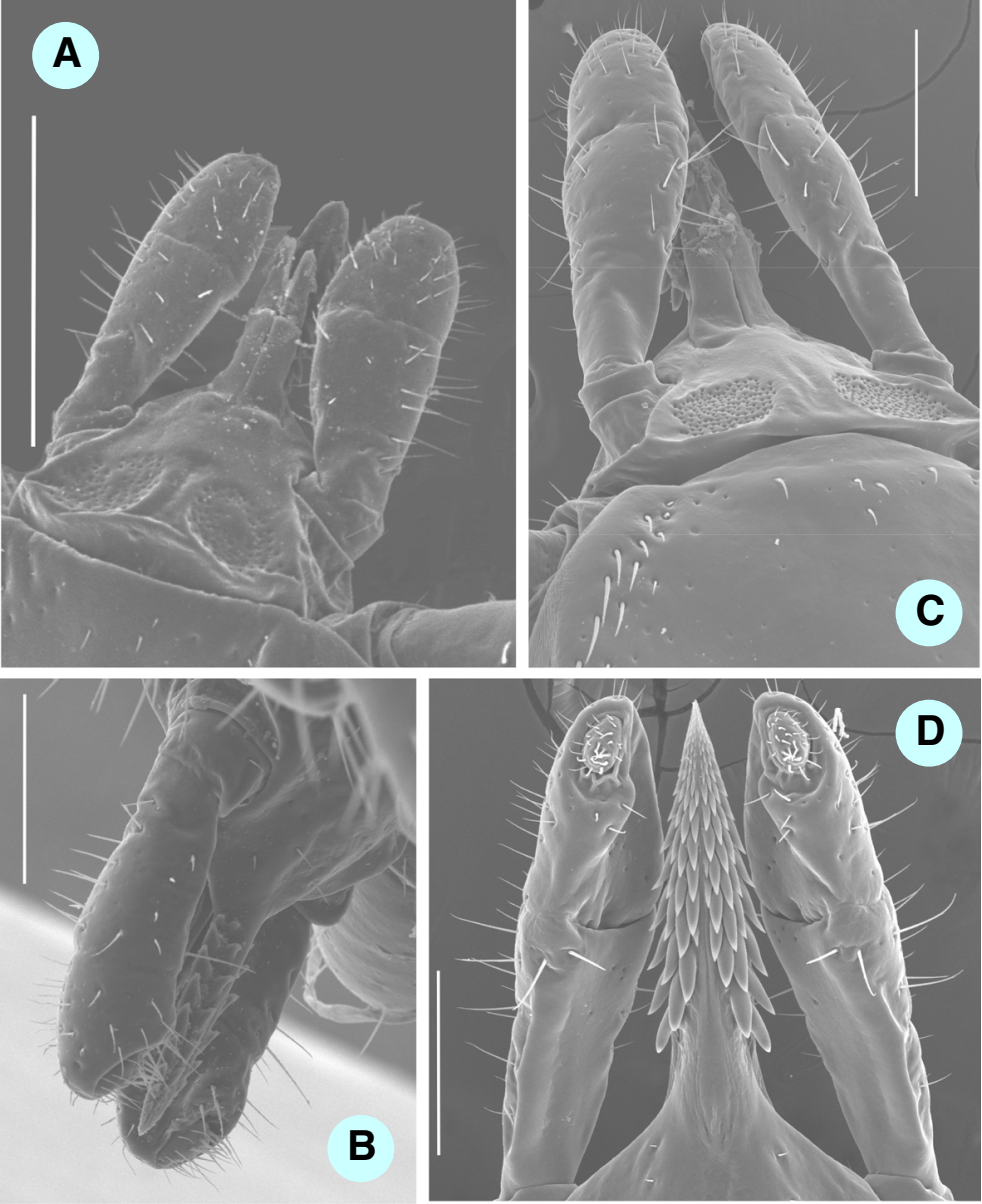
**SEM pictures of head parts of female ticks: engorged *I. ariadnae *** **sp. nov. (A - dorsal, bar = 500 μm; B - ventral, bar = 250 μm) and unfed *I. vespertilionis *** **(C - dorsal, bar = 250 μm; D - ventral, bar = 250 μm).**

**Figure 6:**
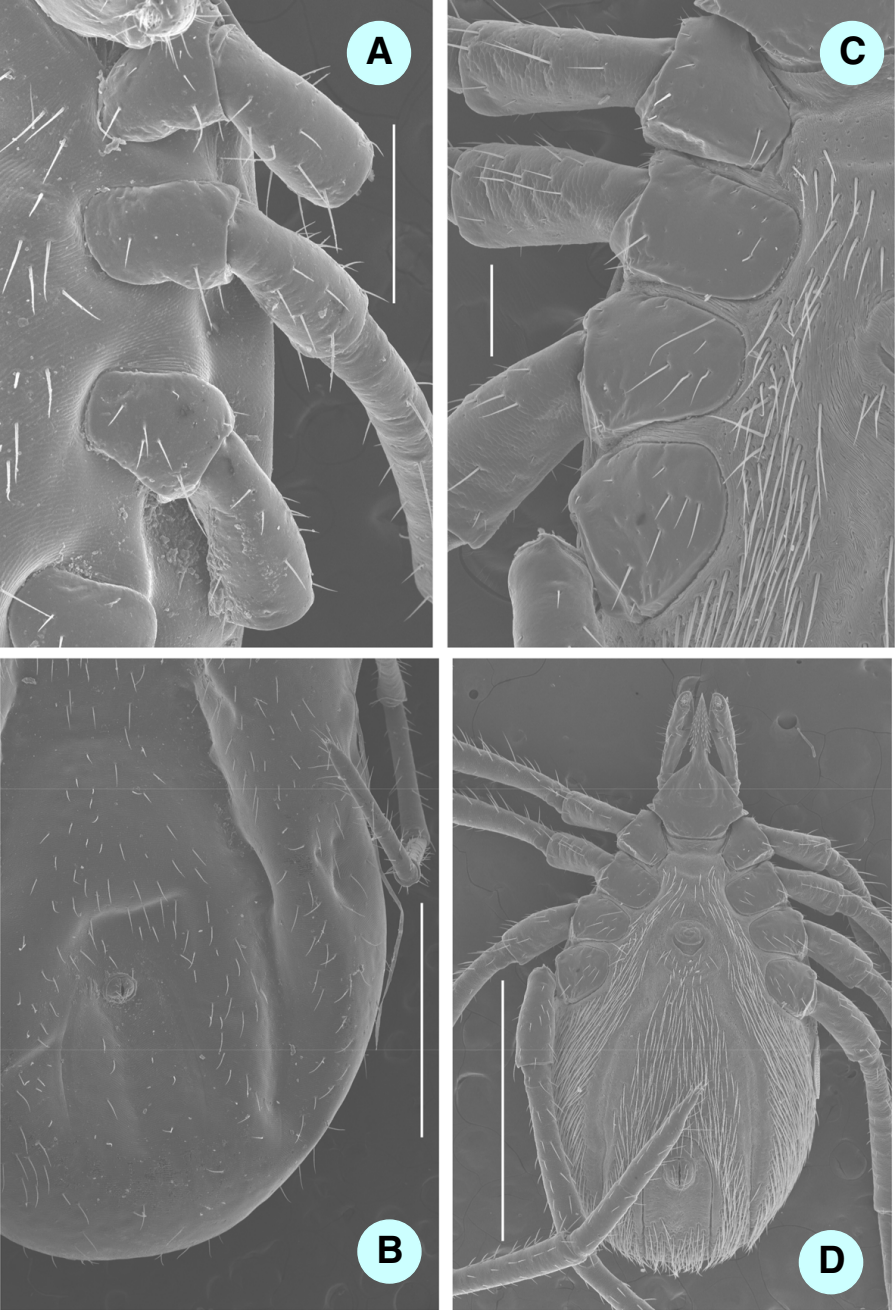
**SEM pictures of ventral parts of female ticks: engorged *I. ariadnae *** **sp. nov. (A - coxae, bar = 500 μm; B - sparse hair covering, bar = 2 mm) and unfed *I. vespertilionis *** **(C - coxae, bar = 250 μm; D - dense hair covering, bar = 2 mm).**

Characteristics of engorged females in comparison with *I. vespertilionis* and *I. simplex* are shown in Table 2, Figures 3, 4, 5, 6.

**Table 2:** **Morphological comparison of *I. vespertilionis*, *I. ariadnae *** **sp. nov. and *I. simplex *** **female**

**Trait**	I. vespertilionis*	***I. ariadnae *** **sp. nov.**	I. simplex*
**Max. engorged size**	10	7	4.5
**Length of legs**	long (tarsus I: 1.6)	long (tarsus I: 1.5)	moderately long (tarsus I: 0.73)
**Basis capituli**	- dorsally posterolaterally flange	- dorsally posterolaterally flange	- dorsally no flange (ventrolaterally triangular ridge)
			- posteriorly sinuous
	- posteriorly straight		
		- posteriorly slightly curved with dorsal ridge	
**Palps (II+III segments)**	long, narrow (0.39 + 0.22)	short, broad (0.28 + 0.16)	short (0.16 + 0.14)
**Joining of II-III segments**	distinct	distinct	indistinct
**Hypostome**	long, 15 rows of slender teeth, ventrally in 6-8 lines	medium, 10 rows of broad teeth, ventrally in 4 lines	short, 8 rows of broad teeth, ventrally in 4-6 lines
**Areae porosae**	large, oval, interval broad	large, oval, interval slightly broad	large, oval, interval narrow
**Scutal setae**	anterolaterally prominent	anterolaterally very few	anterolaterally prominent
**Scutal length per width**	1.76/1.08 (approx. 1.6)	1.15/0.95 (approx. 1.2)	1.13/0.8 (approx. 1.4)
**Idiosoma hair covering**	dense	sparse	dense
**Coxae**	posteroexternally concave, surface flat	posteroexternally convex, surface rounded	posteroexternally concave
**Anal groove**	parallel	posteriorly slightly divergent	posteriorly markedly divergent
**Spiracle opening**	oval, regular outline	oval, irregular outline	(sub)circular, broad

*Descriptions according to Arthur (1956), Babos and Janisch (1958), Nosek and Sixl (1972).

Sizes are given in mm.

#### Gene sequences

Mitochondrial cytochrome oxidase subunit I (COI) gene sequence deposited in the GenBank is KJ490306. Its phylogenetic relationships are shown on Figure 2.

#### Host records and distribution

*Myotis alcathoe* (of two specimens), *Myotis blythii* and *Plecotus auritus*. Distribution is shown on Figure 1.

#### Etymology

The name of the new species refers to Ariadne, the Greek mythological heroine associated with labyrinths. Its relevance to the new species is that most specimens were collected at the caves in the Ariadne Cave system.

#### General

In accordance with section 8.5 of the ICZN’s International Code of Zoological Nomenclature, details of the new species have been submitted to ZooBank with the life science identifier (LSID) zoobank.org:pub: F9432D03-627D-4D86-A307-D9A8E8300361.

## Discussion

Current knowledge on the taxonomy and ecology of *Ixodes* ticks of bats is vague. The review by Arthur [2] notes that descriptions are inadequate in many respects. Data on the seasonal activity of the most widespread species, *I. vespertilionis* have become available only recently [3,14], and these are restricted to ticks collected from bats. At the same time, with growing concern about using molecular methods for the analysis of the taxonomical status of ticks, these were not applied to compare bat ticks, despite the long-known uncertainty in the morphology [2] and nomenclature [15,16] of relevant species. The present study was undertaken to compensate for this lack of information and to contribute to the taxonomy and ecology of ticks that are specialized to bat hosts and have off-host stages living underground, in caves.

Concerning the seasonality of *I. vespertilionis* in caves, tick numbers (including all stages) appeared to be highest in the spring time, in line with what was reported on ticks collected from bats [3,14]. In the present study immature stages were found in all seasons, and this may indicate a continuous, year-round activity in the protected, underground shelters (meaning less exposure to weather variables), with increase or decrease in tick numbers depending on the presence of hosts. Similarly, the blood-sucking of *I. simplex* larvae and nymphs on bats was observed throughout the year, with some seasonal differences [6]. The low number of ticks during the summer time in this study can be explained by hiding (“over-summering”) of stages in the absence of bat hosts in many of the caves, as some bat colonies use different roosts during the winter (i.e. their hibernation) and summer (June: nursing, August-September: mating). Arthur [2] also noted that during the summer time only a few males were reported in caves by some authors. On the other hand, female ticks were not observed on bats during the summer and autumn in another survey [14], but in this study were shown to be present in caves. These females may have developed from nymphs that completed their blood meal in the previous season.

A significant part of the life cycle of ixodid ticks is spent off-host, and in case of *Ixodes* spp. even mating can take place in the environment. Unlike overlapping populations of most ixodid ticks that live on large, continuous land surface biotopes, the small populations and habitats of *I. vespertilionis* in caves are physically well separated, only connected by bat migration. However, because the majority of bats prefer to use the same caves for hibernation repeatedly [17], this may constitute further restrictions for genetic exchange between *I. vespertilionis* populations. Correspondingly, this physical and “host behavioural” separation may entail a high degree of reproductive isolation, a prerequisite for the formation and establishment of divergent genotypes (in the long run speciation).

The present data show that divergent COI genotypes of *I. vespertilionis* exist. Only genotype A and C were identified repeatedly, and only in caves nearby. As genotype A ticks collected at the main collection site were not found in distant caves (which had further, different genotypes), these preliminary results suggest that the occurrence of *I. vespertilionis* genotypes may be associated with particular cave(s). On the contrary, *I. ricinus* isolates of different origin did not show correlation of genotype and geographical location [18]. As concluded from bat ringing data, the three caves of the main collection site and the two westward (where genotype A was found) are connected by bat migration [10,11], thus making the transport of *I. vespertilionis* possible. Bat recaptures in other countries also reflected, that a small interchange of bats between caves close to each other does occur [17]. On the other hand, large distance, mountain ranges or other limiting factors of bat migration may prevent contact (genetic exchange of ticks via bats) between more distant caves.

However, the COI sequences of all four analysed specimens of *I. ariadnae* sp. nov. were identical, although one was collected at a more distant location (Figure 1), reflecting that this tick species has different bat hosts (with different migration habits) from those of *I. vespertilionis*. Hosts of the latter species are mainly *Rhinolophus* species [3], whereas in the present study *I. ariadnae* sp. nov. was collected from three species of other bat genera. At the same time *Miniopterus schreibersii* (the specific host of *I. simplex*) is an unlikely host for *I. ariadnae* sp. nov., because this bat species does not occur in any of the caves where engorged female ticks were collected.

The COI sequence of *I. ariadnae* sp. nov. had only 88% and 86% similarities with *I. vespertilionis* and *I. simplex*, respectively (which showed 85% COI sequence homology with each other). The similarities of COI sequences between tick species of the same genera were estimated to range from 70% to 94% [19]. Accordingly, in comparison with the two hitherto described bat tick species in Europe, the 86-88% sequence similarity of *I. ariadnae* sp. nov. supports its taxonomical status as a separate species. Phylogenetically this species is closer to *I. vespertilionis*, but is placed in a separate cluster, supported by high bootstrap value (Figure 2). Confirming this, its morphology and hosts have also been shown to be different from both *I. vespertilionis* and *I. simplex*.

Further molecular taxonomical studies will be undertaken to investigate the ecology and host associations of this novel species in a broader context.

## Conclusion

*I. vespertilionis* shows year-round activity in caves of Hungary, but larger populations during the autumn, winter and spring and “over-summering” in low numbers. The microenvironment of caves (well separated from each other) appears to support the existence of allopatric genotypes, most likely related to the distance between caves and to bat migration over-bridging certain caves. During the present study a morphologically and genetically distinct new bat tick species was found and is described for the first time. The name *I. ariadnae* sp. nov. is given to this species.

## Competing interests

No competing interests exist.

## Authors’ contributions

SH initiated and supervised the study, processed the samples, extracted the DNA and wrote the manuscript. JK made the electron microscopic pictures and performed phylogenetic analysis. DK, RK, DA, TG and ZSP significantly contributed to the sample collection. ZSK performed molecular analysis and ADM arranged, designed and supervised the Romanian part of the study. All authors read and approved the manuscript.

## References

[B1] Nosek J Sixl W Central-European Ticks (Ixodoidea). Key for determination Jahrbuch der naturwissenschaftlichen Abteilung Joanneum 1972 1/2 61 92

[B2] Arthur DR The Ixodes ticks of Chiroptera (Ixodoidea, Ixodidae) J Parasitol 1956 42 180 196 10.2307/3274734 13320260

[B3] Sevcik M Kristofik J Uhrin M Benda P New records of ticks (Acari: Ixodidae) parasitising on bats in Slovakia Vespertilio 2010 13/14 139 147

[B4] Clifford CM Keirans JE Kohls GM Systematics of the subfamily Ixodinae (Acarina: Ixodidae). 1. The subgenera of Ixodes Ann Entomol Soc Am 1973 66 489 500

[B5] Hornok S Kovács R Meli ML Kontschán J Gönczi E Gyuranecz M Dán Á Molnár V Hofmann-Lehmann R First detection of bartonellae in a broad range of bat ectoparasites Vet Microbiol 2012 159 541 543 10.1016/j.vetmic.2012.04.003 22551590

[B6] Lourenco S Ecology of a host-parasite system. A study in temperate cavedwelling bats 2008 Portugal: PhD Thesis, University of Lisboa

[B7] Piksa K Nowak-Chmura M Siuda K First case of human infestation by the tick Ixodes vespertilionis (Acari: Ixodidae) Int J Acarol 2013 38 1 2

[B8] Hebert PD Ratnasingham S de Waard JR Barcoding animal life: cytochrome c oxidase subunit 1 divergences among closely related species Proc Biol Sci 2003 270 Suppl 1 S96 S99 12952648 10.1098/rsbl.2003.0025 PMC1698023

[B9] Lv J Wu S Zhang Y Chen Y Feng C Yuan X Jia G Deng J Wang C Wang Q Mei L Lin X Assessment of four DNA fragments (COI, 16S rDNA, ITS2, 12S rDNA) for species identification of the Ixodida (Acari: Ixodida) Parasit Vectors 2014 7 93 10.1186/1756-3305-7-93 24589289 PMC3945964

[B10] Kováts D Kurali A Wizl V Kukoda O Surányi D Penszka K Urbányi B Gyenis G First results of the bat research programme in the Danube band Proceedings of the 7th Biological Symposium of the Carpathian Basin (October 13-14, 2011, Budapest) 2011 123 126

[B11] Kováts D Csanádi D Kukoda O Kurali A Ujhegyi N Nursing colonies of the mouse-eared bat (Myotis myotis Borkhausen, 1797) and the lesser mouse-eared bat (M. blythii Tomes, 1857) in underground roosts of the Gerecse and the Visegrádi Mts 2012 Miskolc: International Conference of cave-dwelling bats; SPELEOBATS Hungary

[B12] Hornok S Kováts D Csörgő T Meli ML Gönczi E Hadnagy Z Takács N Farkas R Hofmann-Lehmann R Birds as potential reservoirs of tick-borne pathogens: first evidence of bacteraemia with Rickettsia helvetica Parasit Vectors 2014 7 128 10.1186/1756-3305-7-128 24679245 PMC3976504

[B13] Folmer O Black M Hoeh W Lutz R Vrijenhoek R DNA primers for amplification of mitochondrial cytochrome C oxidase subunit I from diverse metazoan invertebrates Mel Marine Biol Biot 1994 3 294 299 7881515

[B14] Piksa K Górz A Nowak-Chmura M Siuda K The patterns of seasonal activity of *Ixodes vespertilionis* (Acari: Ixodidae) on *Rhinolophus hipposideros* in nursery colonies Ticks Tick Borne Dis 2014 5 69 74 10.1016/j.ttbdis.2013.08.006 24252260

[B15] Babos A Janisch N *Ixodes chiropterorum* sp. n., eine neue Zeckenart in Ungarn Acta Vet Acad Sci Hung 1958 8 389 399

[B16] Beaucourni JC On some palearctic Ixodoidea (Acarina) infesting micro- Chiroptera Ann Parasitol Hum Comp 1966 41 495 502 6008183

[B17] Rivers NM Butlin RK Altringham JD Autumn swarming behaviour of Natterer’s bats in the UK: population size, catchment area and dispersal Biologic Conserv 2006 127 215 226 10.1016/j.biocon.2005.08.010

[B18] Casati S Bernasconi MV Gern L Piffaretti JC Assessment of intraspecific mtDNA variability of European *Ixodes ricinus* sensu stricto (Acari: Ixodidae) Infect Genet Evol 2008 8 152 158 10.1016/j.meegid.2007.11.007 18206426

[B19] Lu X Lin XD Wang JB Qin XC Tian JH Guo WP Fan FN Shao R Xu J Zhang YZ Molecular survey of hard ticks in endemic areas of tick-borne diseases in China Ticks Tick Borne Dis 2013 4 288 296 10.1016/j.ttbdis.2013.01.003 23538111

